# Europium-Doped Sol-Gel SiO_2_-Based Glasses: Effect of the Europium Source and Content, Magnesium Addition and Thermal Treatment on Their Photoluminescence Properties

**DOI:** 10.3390/molecules23071768

**Published:** 2018-07-19

**Authors:** Hussein Fneich, Nathalie Gaumer, Stéphane Chaussedent, Wilfried Blanc, Ahmad Mehdi

**Affiliations:** 1 University of Montpellier, ICGM, CMOS, CNRS UMR 5253, 34095 Montpellier Cedex 5, France; hussein.fneich@etu.umontpellier.fr; 2 Université d’Angers, LPhiA, UPRES EA 4464, 49045 Angers Cedex 01, France; nathalie.gaumer@univ-angers.fr (N.G.); stephane.chaussedent@univ-angers.fr (S.C.); 3 Université Côte d’Azur, InPhyNi, CNRS UMR 7010, Parc Valrose, 06108 Nice, France; wilfried.blanc@unice.fr

**Keywords:** luminescent materials, sol-gel, silica, europium, magnesium, photoluminescence

## Abstract

Rare-earth doped silica-based glasses lead the optical materials due to their tailorable spectroscopic and optical properties. In this context, we took advantage of the sol-gel process to prepare various Eu-doped silica glasses to study their luminescent properties before and after annealing at 900 °C. The effect of magnesium on these properties was studied in comparison with Mg-free-glass. Using TEM, nitrogen sorption, XRD and FT-IR, we confirmed that the magnesium modifies the glass structure and the thermal treatment eliminates the aqueous environment, modifying the structure ordering. The emission spectra and the decay time curves show the advantages of the Mg addition and the annealing on the photoluminescent properties.

## 1. Introduction

Silicate glasses are commonly used as optical materials in a broad range of applications due to their ready availability, relatively simple fabrication and large panel of properties (transparency, chemical durability, etc.) [1]. Their luminescent properties are usually modulated by doping with rare-earth ions. Since their discovery in the last years of the 18th century, rare earth elements have displayed wide applications, especially in photoluminescence [2,3,4,5]. Their exceptional spectroscopic properties coming from their 4f intra-configurational electronic transitions lead to high quantum efficiencies and narrow emission bands [4].

The simplicity of the sol-gel route in glass fabrication, the low temperature processing conditions, the large number of possibilities in material design and the highly concentrated and homogenous doping levels, make it more applied than conventional melt processing [6,7,8]. Then, rare-earth ion-doped sol-gel silicate glasses are considered promising materials for photonics applications such as lasers, photonics or fiber optics [7,9,10,11]. For decades, Eu^3+^ doped and co-doped sol-gel silica glasses have attracted particular interest for their optical properties. In particular, this rare-earth ion exhibits an intense red luminescence of interest for phosphors [3]. This ion is also well known in structural probes used to analyze materials’ matrix structures [12,13,14,15].

Indeed, the Eu^3+^ emission spectrum is composed of several multiplet transitions ^5^D_0_→^7^F*_J_*_= 0,1,…,6_ [16]. The spin-forbidden transitions ^5^D_0_→^7^F_0_ and ^5^D_0_→^7^F_2_ are strongly influenced by the electrostatic local field of the luminescent site. On the contrary, the ^5^D_0_→^7^F_1_ transition is mainly magnetic dipolar and intensity-insensitive to the local field. For these reasons, the analysis of the ^5^D_0_→^7^F_0,1,2_ band shapes can provide relevant information on the local structure around the rare-earth ion [17]. Luminescent properties of rare-earth ions depend on many factors such as rare-earth ion concentrations, impurities (hydroxyl, etc.), atomic arrangement, etc. To improve the luminescent properties of europium ions, several co-dopants have been investigated [18]. Co-doping with aluminum has been extensively studied [19,20]. In this article, we focus our attention on europium-doped sol-gel glasses co-doped with magnesium.

Magnesium oxide, MgO, is miscible in silicate glasses and considered as one of the most important silicate glass network modifiers in order to increase the mechanical strength and chemical stability of glass [21,22], although, once its quantity exceeds 20 mol%, it starts to act as network former or intermediate [22,23,24]. Furthermore, the incorporation of MgO makes the glass network tighter and the network dimensionality lower without phase segregation. It also decreases the glass transition temperature (T_g_) and increases the glass crystallization temperature (T_c_). These actions broaden the glass sintering temperature window (T_c_−T_g_), leading to the inhibition of glass crystallization and enabling the sintering of the glass without crystalline phase formation. These effects are due to the fact the newly formed Si–O–Mg bonds are weaker than Si–O–Si bonds [22,25,26,27,28].

To the best of our knowledge, only one article has dealt with the optical properties of Eu^3+^-doped sol-gel silica glass co-doped with magnesium [29]. This study reported an increase of the luminescence intensity of Eu^3+^ when magnesium was added compared to Mg-free silica, but these conclusions are based on the study of nanoparticles where surface effects can impact the luminescent properties. Moreover, luminescence decay curves were not measured, nor OH content.

In this work, we studied the effect of Eu(III) ions and their concentrations on the photoluminescent properties of doped silicate glass, by using two different salts: europium (III) chloride hexahydrate (EuCl_3_·6H_2_O) and the europium (III) nitrate hexahydrate (Eu(NO_3_)_3_·6H_2_O), with three different molar contents: 0.2, 0.5 and 1 mol%. In addition, magnesium nitrate hexahydrate (Mg(NO_3_)_2_·6H_2_O) with 10 mol% was introduced in the silica matrix and its effect on the photoluminescence properties was studied. Finally, the heat treatment effect on the photoluminescence properties of the materials was investigated by annealing all samples at 900 °C. The thermal treatment of the materials has some significant effects on the silica structure, such as promoting the SiO_2_ condensation in the three-dimensional network which lead to more organized structure after decreasing of the silanol group concentration (Si–OH) [30,31,32,33].

## 2. Results and Discussion

### 2.1. Structural Analysis

#### 2.1.1. Characterization of the Porosity

**Figure 1:**
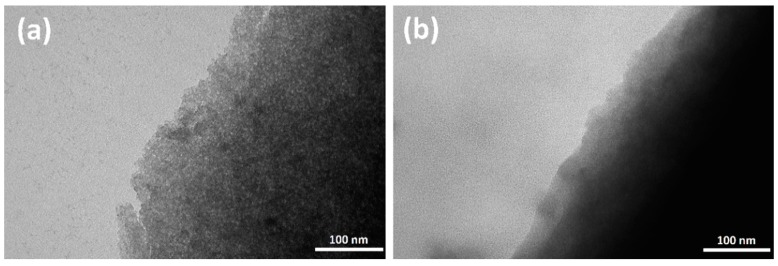
TEM images of $SiEu_{Cl}^{0.2}$ before (**a**) and after (**b**) thermal treatment at 900 °C.

All the samples were characterized by TEM. The TEM images for all samples are similar to those reported in the Figure 1 related to the $SiEu_{Cl}^{0.2}$ sample before and after thermal treatment. Before heat treatment, the texture of the as-prepared SiO_2_ sol-gel powder is heterogeneous in terms of porosity (Figure 1a). The materials obtained after the hydrolysis and poly-condensation of silicon alkoxides are defined as xerogels, where few nm nanoparticles of SiO_2_ are coalesced into larger agglomerates. After annealing at 900 °C, the structure looks more homogeneous (Figure 1b). The powdered microporous xerogel is transformed into a semi-densified silica powder.

To evaluate the change of the porosity, surface area and pore volume were measured by nitrogen sorption analysis (BET method). Results are reported in Table 1.

For all samples, heat treatment leads to a decrease of the BET surface area and the pore volume. While the BET surface areas are similar for all the as-prepared sol-gel glasses, the heat treatment is much more efficient to reduce the BET surface area when the sample contains magnesium. Indeed, in case of Mg-free silica, the surface area reduced after annealing by 36.2% and 53.4% for $SiEu_{Cl}^{1}$ and $SiEu_{N}^{1}$, respectively. This reduction is 82% for $SiMg_{N}^{10}Eu_{N}^{1}$. The same trends are observed for all the europium concentrations.

The incorporation of magnesium into silica tends to modify the structure of the glassy network. Less SiO_4_ tetrahedra are connected, decreasing the viscosity of the glass [34]. This may explain the lower BET surface area observed for Mg-doped samples [35].

Regarding the pore volume, the addition of magnesium increases the pore volume. It is about two times higher compared to Mg-free glass. But, it reduces highly with the heat treatment for all samples. This reduction is 72%, 63% and 68% for $SiEu_{Cl}^{1}$, $SiEu_{N}^{1}$ and $\;SiMg_{N}^{10}Eu_{N}^{1}$ respectively, it proves the samples densification by heat treatment.

#### 2.1.2. X-Ray Diffraction

**Figure 2:**
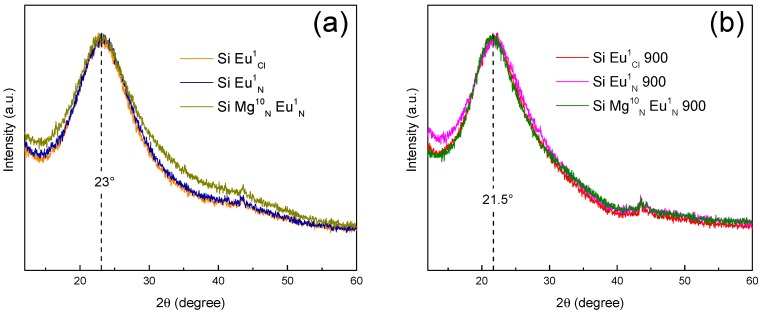
XRD patterns of (**a**) $SiMg_{N}^{x}Eu_{z}^{1}$ (**b**) $SiMg_{N}^{x}Eu_{z}^{1}\;900$.

To study the structural evolution as a function of the temperature, the samples were characterized by X-Ray Diffraction (XRD). XRD patterns of six samples are presented in the Figure 2. For all samples, the patterns contain the same features: (i) a broad band at 2θ ≈ 22° and (ii) peaks at ~44°. The broad band at 2θ ≈ 22° is the fingerprint of the amorphous structure of the network [32,36]. After heat treatment, the position of this peak shifts to lower angle, from 23° to 21.5°. Such shift is usually attributed to a change of the silica network, making evidences of an amorphous structure ordering [32]. The peaks at 2θ ≈ 44° correspond to an artifact, due to the sample holder. We do not observe the presence of α-quartz crystalline phase as reported by Bansal et al., nor Mg(NO_3_)_2_·6H_2_O, which was expected to be present only for nominal concentration higher than 15 mol% [37].

Otherwise, there is no presence of MgO corresponding peaks. Normally, MgO crystal shows three weak diffraction peaks around 2θ = 36.9, 74.6, and 78.6° and two others around 2θ = 42.8 and 62.2° stronger than amorphous silica broad fuzzy diffraction lines [38,39,40].

#### 2.1.3. FT-IR Analyses

**Figure 3:**
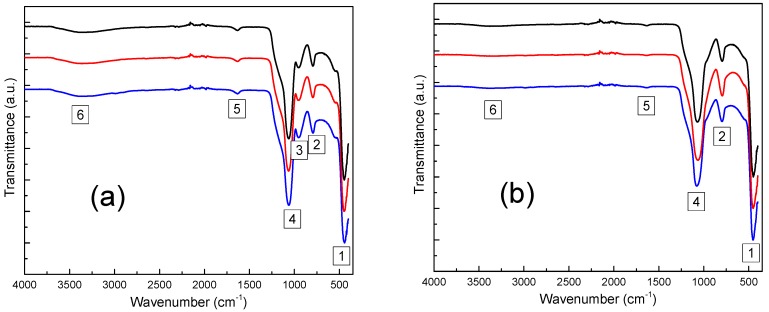

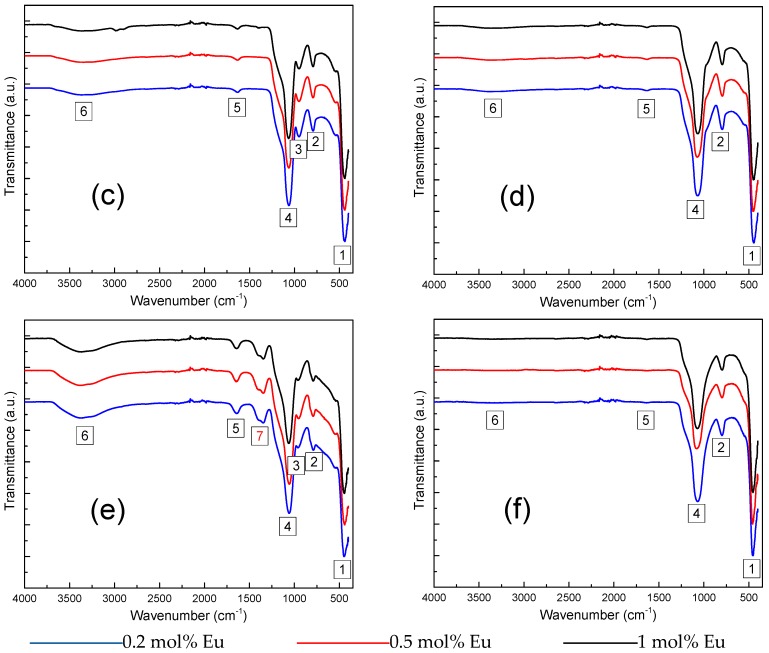
Room temperature FT-IR spectra of (**a**) $SiEu_{Cl}^{y}$ (**b**) $SiEu_{Cl}^{y}\;900$ (**c**) $SiEu_{N}^{y}$ (**d**) $SiEu_{N}^{y}\;900$ (**e**) $SiMg_{N}^{10}Eu_{N}^{y}$ (**f**)$\;SiMg_{N}^{10}Eu_{N}^{y}\;900$.

As fast and non-destructive analysis technique, FTIR spectroscopy is known to detect the local changes and give information about the functional groups of the glass structure. Therefore, we have recorded the FTIR spectra for the studied samples (Figure 3). All these bands can be associated to the SiO_2_ network and the presence of OH groups.

First of all, it is worth presenting some specific bands of IR spectra of the SiO_2_ based compounds and the information they gave. Three vibrational modes of the Si–O–Si group are mainly present in the region between 400 and 1300 cm^−1^. A strong peak near 450 cm^−1^ (labeled “1” in the Figure 3) is assigned to a mode called rocking mode or rocking motion that proves the silicate’s three-dimensional network presence in the glass. The oxygen atom, bridging two adjacent silicon atoms forming siloxanes bonds (Si–O–Si), vibrates perpendicularly to the Si–O–Si plane [41,42,43,44,45,46]. The next one, a weaker band around 800 cm^−1^ (labeled “2”) is associated to a symmetric stretching mode, a vertical motion of the oxygen atoms in the Si–O–Si plane [41,42,43,44,45]. Finally, a strong band around 1100 cm^−1^ (labeled “4”) is assigned to the Si–O–Si asymmetric stretching mode where the bridging oxygen atoms move horizontally in to the Si–O–Si plane [41,42,43,44,45]. In addition, the shoulder at about 1200 cm^−1^ is generally interpreted as a sign of the amorphous network of SiO_2_ [30].

The IR bands associated to the presence of OH group are labeled 3, 5 and 6 in Figure 3. In the spectra of non-annealed materials, the low frequency band around 950 cm^−1^ (“3”) is assigned to the Si–OH symmetric stretching vibrations [43,44,47]. The other low frequency band around 1650 cm^−1^ (“5”) is attributed to the bending vibration of adsorbed molecules of water H_2_O [43,44] which confirm that the broad band between 3000–3650 cm^−1^ (“6”) owes to an overlap of the O–H stretching vibrations in H–bonded molecular water adsorbed by the samples (3300–3500 cm^−1^) and the H stretching of Si–OH groups inside of the glass (around 3650 cm^−1^) [43,44,48].

Before thermal treatment, all the samples exhibit IR bands associated to the presence of OH groups (bands 3, 5 and 6). Comparing the spectra in Figure 3a–d, there are no differences in the positions and shapes of the peaks for the samples with different europium complexes and quantities, both before and after annealing at 900 °C. The samples containing magnesium exhibit higher content of OH groups (Figure 3e). This could be due to the presence of magnesium nitrate which is hexahydrated. After annealing at 900 °C, the OH-related bands decrease strongly and almost disappear.

These results are confirmed by the elementary analysis with 1.76 wt.%, 1.82 wt.% and 2.96 wt.% of hydrogen in $SiEu_{Cl}^{x}$,$\;SiEu_{N}^{x}$ and $SiMg_{N}^{10}Eu_{N}^{x}$ respectively and 1.22 wt.%, 1.19 wt.% and 0.4 wt.% after thermal treatment of the respective materials. Therefore, we noticed a decreasing by 31 wt.%, 34 wt.% and 86 wt.% of hydrogen quantities after annealing of $SiEu_{Cl}^{x}$, $SiEu_{N}^{x}$ and $SiMg_{N}^{10}Eu_{N}^{x}$ respectively. We can observe that the liberation of the OH groups easier after the Mg addition that led to a more open structure seen by the higher pore volume compared to Mg-free-glass. The origin of the residual traces of water in the annealed samples is not clear and could be due to adsorbed water from the ambient atmosphere during the preparation for the different analyses [37].

Finally, a peak between 1350–1400 cm^−1^ (labeled “7”) is present only for the silica containing magnesium before annealing (Figure 3f). It is assigned to the presence of NO_3_^−^ ions in the glassy network [37,49]. This IR peak disappears after annealing at 900 °C, which indicates the decomposition of these ions at high temperature.

### 2.2. Photoluminescence Study

#### 2.2.1. Emission Spectra

**Figure 4:**
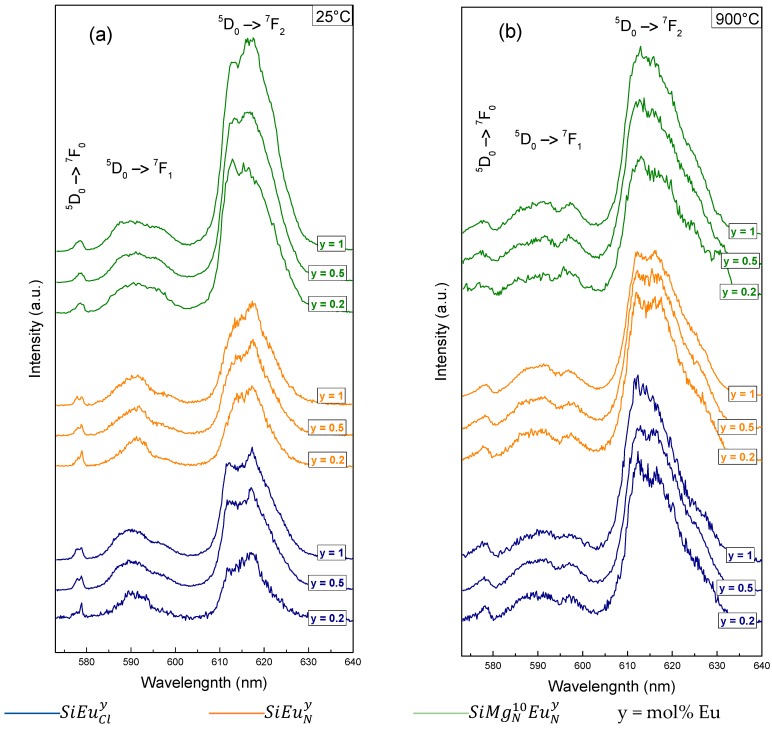
Room temperature emission spectra of the different samples (**a**) before and (**b**) after the thermal treatment at 900 °C (Intensities are normalized to the maximum of the ^5^D_0_→^7^F_1_ emission band).

The Eu^3+^ emission spectrum is composed of several multiplet transitions ^5^D_0_→^7^F*_J_*_= 0,1,…,6_ [16]. The highest energy one, ^5^D_0_→^7^F_0_, is spin-forbidden and only allowed by *J*-mixing effects [16,50]. The shape and the position of the associated spectral band are strongly influenced by the electrostatic local field of the luminescent site. The second transition, ^5^D_0_→^7^F_1_, is mainly magnetic dipolar and intensity-insensitive to the local field, but its splitting feature directly depends on the crystal field experienced by the luminescent site [16,51]. For this reason, the analysis of the ^5^D_0_→^7^F_1_ band shape can provide relevant information on the local structure around the rare-earth ion [17]. The intensity of this band is also used as a reference to normalize and compare different emission spectra. The intensity and the 5-fold splitting of the ^5^D_0_→^7^F_2_ transition both depend drastically on the crystal field strength [16,52]. Its band shape is therefore trickier to analyze and to link with the structure of the luminescent site. Figure 4 displays the emission spectra of the different samples and allows observing the effects of the thermal treatment, of the Eu^3+^ doping concentration, of the Mg co-doping and of the Eu^3+^-salt used in the sol-gel process. All spectra were recorded at room temperature and the intensities were normalized to the ^5^D_0_→^7^F_1_ emission.

Considering samples before the thermal treatment (Figure 4a), the most drastic effect is due to the Mg co-doping. Independently of the Eu^3+^ concentration and for both Eu^3+^ salts, the emission spectra of the Mg-free samples are very similar: the inhomogeneous line broadening is weak for the ^5^D_0_→^7^F_0_ and ^5^D_0_→^7^F_1_ emission bands, which is typical of the luminescent ion in an aqueous environment where the sites are similar and composed of OH groups [53]. On the contrary, one can observe from the emission spectrum of the co-doped sample that Mg allows an inhomogeneous line broadening, even for the lowest Eu^3+^ doping concentration. This result is surprising because it was measured by elementary analysis a hydrogen content before thermal treatment higher in the Mg co-doped sample (2.96 wt.%) than in the Mg-free samples (1.76 wt.% and 1.82 wt.%). It means that a specific structural organization in the Mg co-doped sample prevents OH groups from affecting the Eu^3+^ emission properties. Furthermore, one can compare the symmetry of the luminescent sites in comparing the intensities of the dipolar electric transition ^5^D_0_→^7^F_2_ and of the dipolar magnetic transition ^5^D_0_→^7^F_1_. Indeed, lowering of inversion symmetry leads to a mixing of different parity states, thus allowing electric dipolar transitions. Consequently, low value of the peak intensity ratio *R* = I (^5^D_0_→^7^F_2_)/I (^5^D_0_→^7^F_1_) is indicative of high symmetry sites, whereas high value of *R* is indicative of a distribution of different distorted sites [53]. The maximum intensity is considered as of the highest point of each peak. From spectra of Figure 4a, before thermal treatment, it was calculated *R* values ranging from 2.2 to 3.6 for the Mg-free samples, whereas *R* varies from 5.0 to 6.9 in the Mg co-doped samples. Because an aqueous environment allows high symmetry sites for the luminescent ions [54], these results are indicative of the presence of OH groups around Eu^3+^ in the Mg-free samples. On the contrary, a large diversity of low-symmetry sites in the Mg co-doped samples can be corroborated by large *R* values and the inhomogeneous line broadening observed from the emission spectra.

Whether for thermally treated samples or for untreated ones (Figure 4a,b), no clear trend can be observed on the emission spectra by varying the Eu^3+^ doping concentration from 0.2 to 1 mol%. Only a slight increase of the inhomogeneous line broadening, together with a weak increase of the *R* ratio, can be observed for the higher Eu^3+^ concentration. It means that there are no drastic structural modifications and that an increase of the doping level does not lead to a rare-earth clustering trend (at least up to the highest concentration used in this study) but only to an enhancement of the structural diversity of the luminescent sites.

The use of a nitrate or a chloride europium salt also weakly impacts the emission spectra: all Mg-free samples globally display similar spectra, whether for thermally treated samples or for untreated ones. Only small differences can be observed between spectra of Mg-free samples before the thermal treatment. Nitrate ion is known to easily form europium complexes and it can affect the close environment of the europium ions, especially in making more difficult the solvent removal during the drying stage of the sol-gel process. This can therefore explain the fact that the $SiEu_{N}^{y}$ samples at 25 °C give emission spectra more like those obtained from Eu^3+^ in an aqueous environment. Anyway, the thermal treatment at 900 °C clearly cancels any effect of the europium salt on the emission spectra and therefore on the local environment of the rare-earth ions ($SiEu_{N}^{y}$ and $SiEu_{Cl}^{y}$ samples in Figure 4b).

The effect of the thermal treatment is drastic on the emission spectra for all samples. Normally, the presence of OH groups creates fluorescence quenching due to the non-radiative de-excitation of Eu^3+^ ions caused by the energy transfer to OH vibration, while the heat treatment dehydrate and condensate the glass and lead to better integration of Eu^3+^ ions in the glassy structure and better fluorescence [55]. Especially in Mg-free samples, one can observe an inhomogeneous line broadening together with an increase of the *R* ratio. This behavior is typical of the removal of the solvents and is commonly interpreted as a change from an aqueous environment to a cationic dominated one around the rare-earth ion: luminescent sites become more various and distorted with low symmetry [20]. For the Mg co-doped samples, the thermal treatment enhances the inhomogeneous line broadening that is already observed from the untreated samples. One can therefore suppose that the thermal treatment fully acts on the structure and/or on the structural diversity of the luminescent sites which are already free of solvent. Since at the same time the hydrogen content measured by elementary analysis is highly decreased by the thermal treatment (from 2.96 wt.% to 0.4 wt.%), one can assume that OH groups are not initially located within the close environment of the rare-earth ions in the Mg co-doped samples.

#### 2.2.2. Decay Curves and Lifetimes

**Figure 5:**
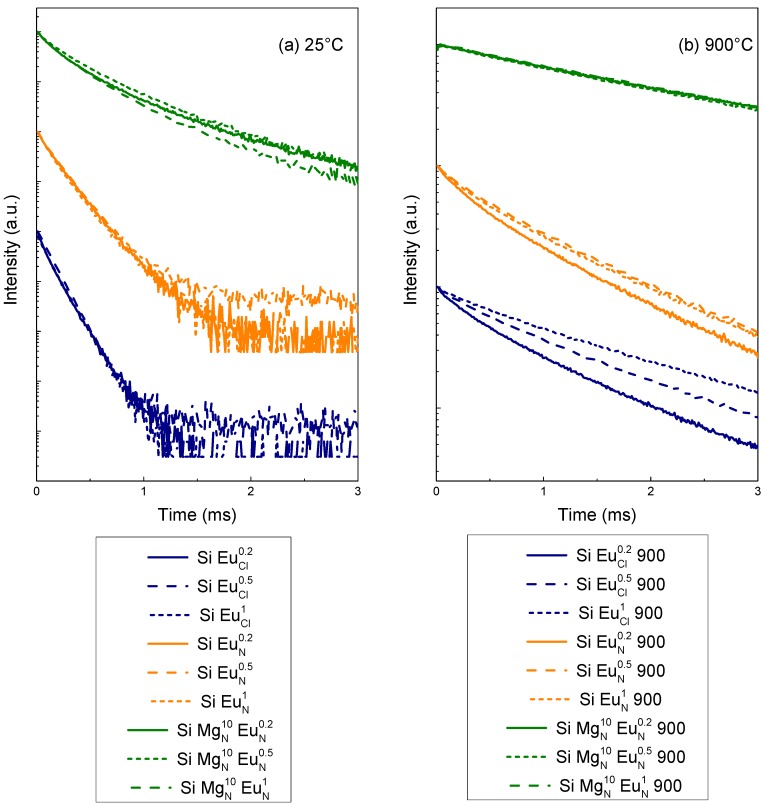
Luminescence decay curves of the ^5^D_0_ level of Eu^3+^ measured at the maximum intensity of the ^5^D_0_→^7^F_2_ emission band (615 nm) for the different samples (**a**) before and (**b**) after the thermal treatment at 900 °C.

The luminescence decay curves of the ^5^D_0_ level were recorded for all samples at the wavelength corresponding to the maximum intensity of the ^5^D_0_→^7^F_2_ emission band. Figure 5 displays the decay curves before and after the thermal treatment at 900 °C.

An important effect of an aqueous environment is the quenching of luminescence of Eu^3+^ ions [20]. In general, the organic residuals have high vibration frequencies and can efficiently quench luminescence of optically active ions. It is reported that the OH vibration frequency can be as high as 3340 cm^−1^ [56] so that only five phonons are needed to bridge the excited state ^5^D_0_ and the ground state ^7^F*_J_* levels of Eu^3+^. As a result, the presence of OH group near Eu^3+^ ions provide a nonradiative relaxation pathway and causes fluorescence quenching. On the other hand, thermal treatment can reduce or eliminate residual hydroxyl and other organic groups in the samples, reducing OH vibration quenching, and consequently increase the decay time. So, the lifetime increases with the thermal treatment at 900 °C for all samples is due to the reduction of the quenching centers.

Except for the $SiMg_{N}^{10}Eu_{N}^{y}$ samples treated at 900 °C, all samples show decay curves that are not single-exponential. These decay curves could be fitted with a 2-exponential curve but the origin of the two lifetime constants is not clear. For this study, we focus our attention on the comparison between all samples. Then, lifetimes were estimated in calculating an average value as:

(1) τ=∫I(t)tdt/∫I(t)dt

where I(t) is the intensity decay profile. Obtained values are reported in Table 2.

Comparing the decay curve profiles (Figure 5) and the average lifetimes (Table 2) for all samples, one can observe that the Eu^3+^ dopant concentration has no significant effect. This is in agreement with the observations made from the emission spectra and it confirms that, up to 1 mol%, there is no concentration quenching of the luminescence which is generally attributed to the conjunction of rare-earth clustering with energy transfers between clustered ions by a cross-relaxation mechanism or phonon-assisted energy transfer [19].

The effect of the europium salt is also weak whether for thermally treated Mg-free samples or for untreated ones. The mean difference between $SiEu_{N}^{y}$ and $SiEu_{Cl}^{y}$ samples is that the thermal treatment is slightly more effective on the lifetime lengthening by using a europium chloride salt: in average, the lifetime is multiplied by about 10 and 6 for $SiEu_{Cl}^{y}$ and $SiEu_{N}^{y}$ samples, respectively. It confirms accordingly that the solvent removal around the rare-earth ions is more difficult by using a nitrate salt.

As observed from the emission spectra, the effect of Mg on the decay curves and lifetimes is striking. Before the thermal treatment, the $SiMg_{N}^{10}Eu_{N}^{y}$ samples provide decay curves which are strongly non-exponential, with corresponding lifetimes three times longer than those obtained from Mg-free samples. In a previous study [20], it has been shown that a progressive increase of the thermal treatment temperature leads to a progressive evolution from a fast almost exponential decay (0.15 ms) to a slower almost exponential decay (1.5 ms) in a Eu^3+^-doped silica gel. In the same study [20], an Al codoping together with a progressive thermal treatment allowed to reach longer lifetimes (up to 2.2 ms at 800 °C) associated with non-exponential decay curves. In the present study, without any thermal treatment, the Mg co-doping allows also a lengthening of the lifetime (from 0.15 ms to 0.42 ms) and leads also to non-exponential decay curves. This can be considered as an additional evidence of OH groups that are initially rarely located within the close environment of the rare-earth ions in the Mg co-doped samples. The thermal treatment at 900 °C of the Mg co-doped samples completes the solvent removal and provides luminescent sites with reduced nonradiative relaxation pathway since the lifetimes are about 80% longer than in the Mg-free samples.

## 3. Materials and Methods

### 3.1. Samples Preparation

**Figure 6:**
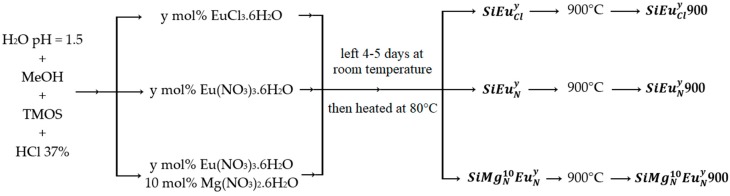
The main preparation route of the different glasses. The bolded names correspond to the nomenclature of the samples.

Europium and magnesium doped silica bulks were synthesized by sol-gel process (inorganic polymerization). A summary of the preparation method with the final materials names are presented in Figure 6.

First, appropriate amounts of EuCl_3_·6H_2_O, Eu(NO_3_)_3_·6H_2_O and Mg(NO_3_)_2_·6H_2_O, purchased from Sigma-Aldrich (Saint-Quentin-Fallavier, France), were dissolved in 5 mL of acidic water with pH = 1.5. It worth noting that the acidic water (pH = 1.5) was prepared by placing 2.4 mL of HCl (37%, density 1.3 g/mL) in a 1 L Erlenmeyer flask which had been filled with ultra-pure water in order to obtain an acidic solution with [H^+^] = 0.0315 mol/L, where the pH = −log (0.0315) = 1.5. After their total dissolution, 5 mL of methanol (CH_3_OH) were added, followed by 3.64 g (0.024 mol) of tetramethylorthosilicate (TMOS). The obtained mixture was stirred for 10–15 min at room temperature to hydrolyze the SiOMe into SiOH groups. Then, 4–5 drops of 37% hydrochloric acid (HCl) were added in order to decrease the pH permitting the polymerization take place. After stirring for 6 h, the mixture was left for several days under air at room temperature until obtaining dry pieces of glasses, which were grinded in a fine white powder and heated to 80 °C under air for 18 h to make sure of the total evaporation of all solvents. In order to study the effect of the thermal treatment, the half of the obtained powder of each sample was heated to 900 °C for 5 h using a heating rate of 10 °C/min. The different starting compositions of the studied materials are given in Table 3.

The main nomenclature is $SiMg_{z}^{x}Eu_{z}^{y}$ where “x” and “y” are the molar percentages of the magnesium and europium, respectively, and “z” is the salt source (“N” for nitrate and “Cl” for chloride). As an example, the $SiMg_{N}^{10}Eu_{N}^{0.2}$ contains 89.8 mol% SiO_2_, 10 mol% MgO (from Mg(NO_3_)_2_·6H_2_O) and 0.2 mol% Eu_2_O_3_ (from Eu(NO_3_)_3_·6H_2_O).

### 3.2. Characterization Techniques

Transmission electron microscopy (TEM) images were taken using a 1200EX2 electron microscope operating (JEOL, Tokyo, Japan) at an acceleration tension of 100 kV equipped with an EMSIS camera mounted with an 11-megapixel CCD sensor (EMSIS GmbH, Muenster, Germany).

N_2_ Physisorption experiments were carried out at −196 °C on a TriStar 3000 instrument (Micromeritics, Norcross, GA, USA). Samples were outgassed under vacuum at 150 °C over-night. Equivalent BET specific surface areas were determined in the relative pressure range *P*/*P_0_* from 0.01 to 0.3 using 74 points. The total pore volume was measured at *P*/*P_0_* > 0.985.

X-Ray Diffraction (XRD) experiments were performed using an X’Pert MPD θ-θ diffractometer (Philips, Amsterdam, Netherlands) with Cu Kα radiation (λ = 1.5418 Å). It is equipped with the X’Celerator detector and nickel filter. Analyses were carried out between 12°–60° as interval with a step size of 0.033.

Fourier-Transform Infra-Red (FTIR) analysis was carried out in the range 400–4000 cm^−1^ using a Spectrum Two™ spectrometer (Perkin Elmer, Waltham, MA, USA) in Attenuated Total Reflectance (ATR) mode. The spectra were obtained from the accumulation of 10 scans with a used resolution of 2 cm^−1^.

Elementary analysis was carried out with a Vario Micro Cube (Elementar, Munich, Germany) equipped with a UMX5 Comparator (Mettler Toledo, Zurich, Switzerland) balance with a 0.1 µg precision. The samples were combusted at 1150 °C to form an H_2_O gas. The mass percentages measurement of hydrogen is detected by a thermal conductivity detector (TCD) katharometer (Elementar Americas Inc., Ronkonkoma, NY 11779, USA).

A pulsed doubled Nd:YAG laser (Spectra-Physics, Santa Clara, CA, USA) emitting at 532 nm with 10 Hz frequency was used as excitation source for the photoluminescence and lifetime measurements of europium. All measurements were performed with a photon counting device (Stanford Research Systems, Sunnyvale, CA, USA) at room temperature. The decay curves were carried out around the maximum of the emission line of europium with temporal window width of 10.24 μs.

## 4. Conclusions

In this article, the influence of Eu content and precursor, and the thermal treatment on the photoluminescent properties of Eu^3+^ were studied. Aluminum was used in many works to improve the luminescent properties of rare-earth ions in silica-based materials, but we focus our attention on an alternative element, magnesium. Mg-free samples have similar luminescent properties for all the europium concentrations. The annealing at 900 °C of europium-doped silica glass increases fluorescence lifetimes by removing aqueous sites and modifying the crystal field. Generally, the addition of magnesium is beneficial for luminescent properties. Both in the xerogel and in the calcined material, lifetime is improved by adding magnesium, despite a higher content of OH groups. Calcination of Mg-glass leads to a highly radiative material with a longer lifetime.

## Figures and Tables

**Table 1:** BET surface area and pore volume for representative samples before and after annealing.

Sample	BET Surface Area m^2^/g	Pore Volume cm^3^/g
$SiEu_{Cl}^{1}$	334.0 ± 1.1	0.321
$SiEu_{Cl}^{1}900$	212.9 ± 1.7	0.091
$SiEu_{N}^{1}$	306.9 ± 2.0	0.218
$SiEu_{N}^{1}900$	142.8 ± 1.1	0.082
$SiMg_{N}^{10}Eu_{N}^{1}$	289.6 ± 2.3	0.519
$SiMg_{N}^{10}Eu_{N}^{1}900$	52.4 ± 1.4	0.166

**Table 2:** Average lifetime τ of the ^5^D_0_ level for the different samples before and after thermal treatment at 900 °C.

Samples		τ (ms)	
		25° C	900 °C
$SiEu_{Cl}^{y}$	*y* = 0.2 mol%	0.11	1.03
	*y* = 0.5 mol%	0.12	1.42
	*y* = 1.0 mol%	0.13	1.22
$SiEu_{N}^{y}$	*y* = 0.2 mol%	0.16	0.87
	*y* = 0.5 mol%	0.16	0.98
	*y* = 1.0 mol%	0.15	0.96
$SiMg_{N}^{10}Eu_{N}^{y}$	*y* = 0.2 mol%	0.40	1.80
	*y* = 0.5 mol%	0.42	1.80
	*y* = 1.0 mol%	0.34	1.78

**Table 3:** Starting composition and nomenclature of mixed oxide.

Sample	EuCl_3_·6H_2_O mol%	Eu(NO_3_)_3_·6H_2_O mol%	Mg(NO_3_)_2_·6H_2_O mol%	TMOS mol%
$SiEu_{Cl}^{0.2}$	0.2	-	-	99.8
$SiEu_{Cl}^{0.5}$	0.5	-	-	99.5
$SiEu_{Cl}^{1}$	1	-	-	99
$SiEu_{N}^{0.2}$	-	0.2	-	99.8
$SiEu_{N}^{0.5}$	-	0.5	-	99.5
$SiEu_{N}^{1}$	-	1	-	99
$SiMg_{N}^{10}Eu_{N}^{0.2}$	-	0.2	10	89.8
$SiMg_{N}^{10}Eu_{N}^{0.5}$	-	0.5	10	89.5
$SiMg_{N}^{10}Eu_{N}^{1}$	-	1	10	89
